# Antimicrobial Properties and Membrane-Active Mechanism of a Potential α-Helical Antimicrobial Derived from Cathelicidin PMAP-36

**DOI:** 10.1371/journal.pone.0086364

**Published:** 2014-01-21

**Authors:** Yinfeng Lv, Jiajun Wang, He Gao, Zeyun Wang, Na Dong, Qingquan Ma, Anshan Shan

**Affiliations:** Laboratory of Molecular Nutrition and Immunity, Institute of Animal Nutrition, Northeast Agricultural University, Harbin, Heilongjiang, China; Aligarh Muslim University, India

## Abstract

Antimicrobial peptides (AMPs), which present in the non-specific immune system of organism, are amongst the most promising candidates for the development of novel antimicrobials. The modification of naturally occurring AMPs based on their residue composition and distribution is a simple and effective strategy for optimization of known AMPs. In this study, a series of truncated and residue-substituted derivatives of antimicrobial peptide PMAP-36 were designed and synthesized. The 24-residue truncated peptide, GI24, displayed antimicrobial activity comparable to the mother peptide PMAP-36 with MICs ranging from 1 to 4 µM, which is lower than the MICs of bee venom melittin. Although GI24 displayed high antimicrobial activity, its hemolytic activity was much lower than melittin, suggesting that GI24 have optimal cell selectivity. In addition, the crucial site of GI24 was identified through single site-mutation. An amino acid with high hydrophobicity at position 23 played an important role in guaranteeing the high antimicrobial activity of GI24. Then, lipid vesicles and whole bacteria were employed to investigate the membrane-active mechanisms. Membrane-simulating experiments showed that GI24 interacted strongly with negatively charged phospholipids and weakly with zwitterionic phospholipids, which corresponded well with the data of its biological activities. Membrane permeabilization and flow cytometry provide the evidence that GI24 killed microbial cells by permeabilizing the cell membrane and damaging membrane integrity. GI24 resulted in greater cell morphological changes and visible pores on cell membrane as determined using scanning electron microscopy (SEM) and transmission electron microscope (TEM). Taken together, the peptide GI24 may provide a promising antimicrobial agent for therapeutic applications against the frequently-encountered bacteria.

## Introduction

The discovery of antibiotics effectively reduces the happening of infectious diseases and saved countless lives in less than nine decades. However, the widespread and often indiscriminate use of antibiotics in recent years has led to the rapid emergence of multidrug-resistant “superbug” strains, making infectious diseases increasingly difficult to control with the existing classes of antibiotics. Therefore, there is an urgent need to develop new classes of antimicrobial agents. Data from both the laboratory and the clinic in the last decade indicate that antimicrobial peptides (AMPs) are suitable templates for an alternative class of potential therapeutics [1]. AMPs found in a large number of species constitute a major component of the innate immune system, and they have broad-spectrum activities against gram-negative and gram-positive bacteria [2], including antibiotic-resistant bacterial strains [3] and some fungi [4], viruses [5], parasites [6], and even cancer cells [7]. Moreover, unlike conventional antibiotics that inhibit specific biosynthetic pathways such as cell wall or protein synthesis, the majority of AMPs carry out their respective functions via the rapid physical disruption of microbial cell membranes to cause leakage of cell contents leading to cell death [1]. This is expected to provide an inherent advantage for AMPs in the clinical setting because it is metabolically ‘costlier’ for most microbial to promote resistance by mutating or repairing its membrane components [8]. Currently, there are at least four different commonly used models describing possible AMP membrane-active mechanism that include ‘barrel-stave’, ‘carpet’, ‘toroidal-pore’, and ‘aggregate channel’ models [9], [10].

Although the primary and secondary structures of AMPs display a large heterogeneity, comparison of AMPs sequences reveals that two types of side chains are essential for antimicrobial activity. The cationic side chains provided electrostatic interactions between peptides and the negatively charged membranes and/or cell walls of bacteria, including lipopolysaccharide (LPS) [11]. Nonpolar side chains presumably provided lipophilic anchors that ultimately induce membrane disruption [12]. According an updated database (APD: http://aps.unmc.edu/AP/main.php), AMPs generally possess 1–9 positively charged lysine or arginine residues and up to 50% hydrophobic amino acids. Despite the clear potential of AMPs, only very few AMPs such as polymyxins and gramicidins are being used clinically. The usage of AMPs is mainly limited by systemic toxicities, *in vivo* stability, and high cost for large scale manufacturing [13]. So, truncation and redesign of nature antimicrobial peptides are considered to be a simple and effective approach of developing new antibacterial agents. Recently, Paulsen et al reported that the N-terminal fragment arasin 1(1–23) was almost equally active to the full length peptide arasin 1(a 37-residue peptide) [14]. We have previously shown that the truncation of the C-terminal region of linear chicken-defensin-4 (AvBD-4) retained the antimicrobial activity and eliminated its hemolytic activity [15], [16].

Cathelicidins, a prominent family of antimicrobial peptides, have been identified in many species [17]. Until now, 11 porcine cathelicidins have been found. Among all the porcine cathelicidins, porcine myeloid antimicrobial peptide-36 (PMAP-36) has the highest net positive charge, the proportion of cationic amino acids reach 36% [18]. The high net charge may be advantageous to PMAP-36 binding the bacterial via electrostatic interactions between the positive charged of peptides and the negatively charged molecules at the surface of the bacterial cell membrane. The N-terminal of PMAP-36 is charge-rich domain. Furthermore, structure analyses of this N-terminal region have demonstrated that the highly cationic sequence adopts a typical amphipathic α-helical conformation [18], [19], which provided a good template for researching quantitative structure-activity relationships (QSARs). Previous studies have indicated that the N-terminal α-helical domain of PMAP-36 was its active region, and derivative PMAP-36 (1–20) had the ability to interact and penetrate the bacterial membrane [18], [19].

So, in this study, a series of derivatives were designed and synthesized by truncating of PMAP-36 based on its structural characteristics and residue distribution. The *in vitro* antimicrobial and hemolytic activities of the peptides were determined. Tryptophan fluorescence experiment was performed for the W-containing peptides in the presence of synthetic lipid vesicles to preliminary elucidate the peptide-membrane selectivity, and explained the reason of high bactericidal activity and low toxicity of peptides. Then, whole bacteria were further employed to investigate potential membrane destruction mechanisms. Scanning electron microscopy (SEM) and transmission electron microscopy (TEM) assays allowed us to directly observe the change of cell morphology and the integrity of cell membrane after peptide treatment.

## Materials and Methods

In the present study, written informed consent was obtained from all participants.

### Peptide Design and Sequence Analysis

**Figure 1:**
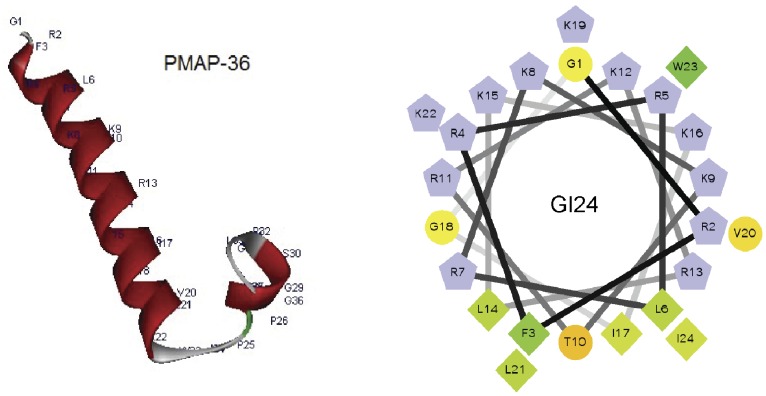
Three-dimensional structure projections of PMAP-36 (left) and helical wheel projections of GI24 (right). In the picture of helical wheel projections of GI24, by default the output presents the hydrophilic residues as circles, hydrophobic residues as diamonds, and potentially positively charged as pentagons. Hydrophobicity is color coded as well: the most hydrophobic residue is green, and the amount of green is decreasing proportionally to the hydrophobicity, with zero hydrophobicity coded as yellow. Hydrophilic residues are coded red with pure red being the most hydrophilic (uncharged) residue, and the amount of red decreasing proportionally to the hydrophilicity. The potentially charged residues are light blue.

According to the three-dimensional structure and helical wheel projection, we designed a series of derivatives by truncating and residue substituting of PMAP-36 (Figure 1). Firstly, different peptide fragments derived from PMAP-36 sequence was obtained. The 24-residue peptide, GI24 contained the entire α-helical region and all of the cationic amino acids of PMAP-36. The helical wheel projection for GI24 indicated that it displays amphipathic residue arrangement with an unusually wide and cationic polar face (Figure 1). So, secondly, GI24-V3 and GI24-V6 were further designed by replacing 3 and 6 cationic residues (R or K) of GI24 with V, respectively, and the antimicrobial activity of GI24-V3 and GI24-V6 is expected to enhance with increased nonpolar face. In addition, the W-substituted analogs of GI24 were designed by replacing the W with different types of amino acids to investigate the contribution of W at position 23 of GI24 on the antimicrobial activity.

The mean hydrophobicity of the peptides was calculated online using CCS scale (http://www.bbcm.univ.trieste.it/~tossi/HydroCalc/HydroMCalc.html). Primary sequence analysis of the peptides was performed by bioinformatics programs ProtParam (ExPASy Proteomics Server: http://www.expasy.org/tools/protparam.html). The three-dimensional structure projection was predicted online by I-TASSER (http://zhanglab.ccmb.med.umich.edu/I-TASSER/). The helical wheel projection was calculated online using the Helical Wheel Projections (http://rzlab.ucr.edu/scripts/wheel/wheel.cgi) [20].

### Peptide Synthesis

The peptides listed in Table 1 were synthesized at GL Biochem Corporation (Shanghai, China) by solid-phase methods using N-(9-fluorenyl) methoxycarbonyl (Fmoc) chemistry. The peptides were amidated at the C-terminal. Reverse-phase high-performance liquid chromatography (HPLC) determined the purity of the peptides to be >95%. Electrospray mass spectrometry was used to identify the peptides. Peptides were then dissolved in DI water at a concentration of 2.56 mM and stored at −20°C before subsequent assessments.

**Table 1:** Amino acid sequences, molecular weights, net charges, and the hydrophobicity values of the peptides.

Peptide	Sequence	Mw^a^	Charge	H^b^
PMAP-36	GRFRRLRKKTRKRLKKIGKVLKWIPPIVGSIPLGCG-NH_2_	4156.27	+14	−1.41
GI24	GRFRRLRKKTRKRLKKIGKVLKWI-NH_2_	3064.93	+14	−2.81
GK12	GRFRRLRKKTRK-NH_2_	1601.00	+9	−5.51
RI12	RLKKIGKVLKWI-NH_2_	1480.96	+6	−0.11
PG12	PPIVGSIPLGCG-NH_2_	1108.37	+1	1.4
GI24-V3	G***V***FRRLRK***V***TRK***V***LKKIGKVLKWI-NH_2_	2921.78	+11	−1.05
GI24-V6	G***V***FR***V***LRK***V***TR***VV***LK***V***IGKVLKWI-NH_2_	2806.64	+8	0.69
GI24-W23A	GRFRRLRKKTRKRLKKIGKVLK***A***I-NH_2_	2949.80	+14	−3.26
GI24-W23K	GRFRRLRKKTRKRLKKIGKVLK***K***I-NH_2_	3006.88	+15	−3.63
GI24-W23L	GRFRRLRKKTRKRLKKIGKVLK***L***I-NH_2_	2991.38	+14	−2.81

a Mw: molecular weight in Daltons.

b H: the hydrophobicity per residue of peptides calculated using CCS scale.

### Antimicrobial Assays

The antimicrobial activity of each peptide was determined against the following bacteria strains: *Escherichia coli* ATCC 25922, *Escherichia coli* UB1005, *Salmonella enterica* serovar typhimurium C77-31, *Staphylococcus aureus* ATCC 29213, *Staphylococcus aureus* ATCC 25923 and *Staphylococcus epidermidis* ATCC 12228. The minimal inhibitory concentrations (MICs) of the peptides were measured according to a modified version of the National Committee for Clinical Laboratory Standards (NCCLS) broth microdilution method as described previously [21]. Briefly, mid-logarithmic phase bacteria were cultured in Mueller-Hinton (MH) broth and then diluted to 1×10^5 ^CFU/ml. Equal volumes (50 µl) of microorganism solution and 2-fold serially diluted different concentrations (0.25–128 µM) peptides in 0.01% (v/v) acetic acid and 0.2% (w/v) bovine serum albumin (BSA, Sigma) were added to each well of the sterile 96-well plate. After incubation for 24 h at 37°C, MICs were determined as the lowest concentration of peptides that prevented visible turbidity by visual inspection. The tests were performed in triplicate. Cultures without peptides and uninoculated MH broth were employed as positive and negative controls, respectively. In addition, the salt stability of the peptides was tested in the MIC assay mentioned above. Different concentrations of monovalent and divalent cations tested were listed in Table S1.

### Measurement of Hemolytic Activity

The hemolytic activity of the peptides was measured as the amount of hemoglobin released by the lysis of human erythrocytes [22]. Briefly, 1 ml of fresh human blood cells (hRBCs) obtained from a healthy donor (Na Dong, Harbin, China) in a polycarbonate tube containing heparin was centrifuged at 1,000×g for 5 min at 4°C [16]. The experimental protocol was reviewed and approved by the ethics committee of the Northeast Agricultural University Hospital. The erythrocytes obtained were washed three times with phosphate-buffered saline (PBS) solution (pH 7.2) and resuspended in PBS. A 50 µl volume of the erythrocyte solution was incubated with 50 µl of various peptides dissolved in PBS for 1 h at 37°C. Intact erythrocytes were pelleted by centrifugation at 1,000×g for 5 min at 4°C, and the supernatant was transferred to a new 96-well plate. The release of hemoglobin was monitored by measuring the absorbance at 570 nm. As negative and positive controls, hRBCs in PBS without the peptide and 0.1% Triton X-100 were employed, respectively.

### Tryptophan Fluorescence and Quenching

Small unilamellar vesicles (SUVs) were prepared for tryptophan fluorescence experiments as described previously [23]. The SUVs, which included egg yolk L-α-phosphatidylethanolamine (PE), egg yolk L-α-phosphatidyl-DL-glycerol (PG), egg yolk L-α-phosphatidylcholine (PC), and cholesterol, were obtained from Sigma-Aldrich Corporation (St. Louis, MO). PE/PG (7∶3, w/w) or PC/cholesterol (10∶1, w/w) lipids were dissolved in chloroform solvents and dried with a stream of nitrogen to form a thin lipid film. The lipid films were resuspended in 10 mM Tris-HCl buffer (10 mM Tris, pH 7.4, 150 mM NaCl, 0.1 mM EDTA) via vortex mixing. The resultant suspensions were sonicated in an ice bath for about 20 min using an ultrasonic cleaner until the solutions clarified.

Tryptophan fluorescence measurements were obtained using an F-4500 fluorescence spectrophotometer (Hitachi, Japan). The fluorescence was excited at 280 nm and emission was scanned at wavelengths ranging from 300 to 400 nm. Spectra of each peptide with liposomes were baseline corrected by subtracting blank spectra of the corresponding solutions without the peptide. Measurements were performed for each peptide in 10 mM Tris-HCl buffer (pH 7.4) with 500 µM PE/PG or PC/cholesterol lipids. The peptide/lipid molar ratio was 1∶50.

Fluorescence quenching experiments were conducted by using an excitation wavelength of 295 nm instead of 280 nm [24]. Tryptophan fluorescence was quenched by titration with acrylamide (Sigma) from a 4 M stock solution to a final concentration of 0.4 M in the absence or presence of liposomes at a peptide/lipid molar ratio of 1∶50. The quenching data were analyzed by the Stern-Volmer quenching constant (*K_SV_*), which was estimated using the Stern-Volmer equation: *F_0_/F* = 1+*K_SV_* (*Q*), where *F_0_* and *F* are the fluorescence values of the peptides in the absence or the presence of acrylamide, *K_SV_* represents the Stern-Volmer quenching constant, and *Q* represents the concentration of acrylamide.

### Outer Membrane Permeabilization Assay

The outer membrane permeability of the peptides was determined by using the N-phenyl-1-napthylamine (NPN) uptake assay as previously described [25]. Briefly, *E. coli* UB1005 were washed and resuspended in buffer (5 mM HEPES, 5 mM glucose, pH 7.4). NPN was added to 2 ml of cells in a quartz cuvette to give a final concentration of 10 µM, and the background fluorescence was recorded (excitation λ = 350 nm, emission λ = 420 nm). Changes in fluorescence were recorded using an F-4500 fluorescence spectrophotometer (Hitachi, Japan). Peptide samples were added to the cuvette, and fluorescence was recorded as a function of time until no further increase in fluorescence was observed. As the outer membrane permeability increased due to the addition of peptide, NPN incorporated into the membrane resulted in an increase in fluorescence. Values were converted to % NPN uptake using the equation: % NPN uptake = (*F_obs_*−*F_o_*)/(*F_100_*−*F_o_*)×100, where *F_obs_* is the observed fluorescence at a given peptide concentration, *F_0_* is the initial fluorescence of NPN with *E. coli* cells in the absence of peptide, and *F_100_* is the fluorescence of NPN with *E. coli* cells upon addition of 10 µg/ml Polymyxin B (Sigma). Polymyxin B is used as a positive control because of its strong outer membrane permeabilizing properties.

### Inner Membrane Depolarization Assay

The cytoplasmic membrane depolarization activity of the peptides was measured by using *E. coli* UB1005 and the membrane potential sensitive fluorescent dye diSC_3_(5) as described previously [26]. Briefly, mid-logarithmic phase *E. coli* were washed with 5 mM sodium HEPES buffer, pH 7.4, containing 20 mM glucose, and resuspended to an OD_600_ of 0.05 in the same buffer. The cell suspension was incubated with 0.4 µM diSC_3_(5) until a stable reduction of fluorescence was achieved (approximately 1 h). Then KCl was added to a final concentration of 0.1 M to equilibrate the cytoplasmic and external K^+^. Two milliliters of the cell suspension were placed in a 1 cm cuvette, and the peptides were added to achieve the desired concentrations. Changes in fluorescence were recorded using an F-4500 fluorescence spectrophotometer (Hitachi, Japan) with an excitation wavelength of 622 nm and an emission wavelength of 670 nm. 0.1% Triton X-100 was employed as positive controls [3].

### FACScan Analysis

The integrity of the bacterial cell membranes after peptide treatment was determined using a previously described method [27]. In brief, mid-logarithmic phase *E. coli* ATCC 25922 were harvested by centrifugation and washed three times with 10 mM PBS solution. The peptides (1×MIC and 2×MIC) were added and incubated for 30 min at 28°C with constant shaking at 140 rpm, and then, propidium iodide (PI, 10 µg/ml final concentration, Sigma) was added and incubated for a further 30 min at 4°C. After incubation, the unbound dye was removed via excessive washing of cells with PBS. Flow cytometry analysis was conducted using a FACScan instrument (Becton–Dickinson, San Jose, CA). *E. coli* cells were incubated with PI without peptide treated as negative control.

### Scanning Electron Microscopy (SEM)

*E. coli* ATCC 25922 were cultured in Mueller-Hinton (MH) broth to mid-log phase and harvested by centrifugation at 1,000×g for 10 min. Cells pellets were washed twice with 10 mM PBS and resuspended to an OD_600_ of 0.2. The cell suspension was incubated at 37°C for 60 min with different peptides at a concentration of 1×MIC. After incubation, the cells were centrifuged and washed 3 times at 5,000×g for 5 min with PBS. Bacterial pellets were then fixed in 500 µl of 2.5% (v/v) glutaraldehyde in PBS at 4°C overnight. Thereafter, the bacteria were washed twice with PBS and dehydrated through a graded ethanol series (50%, 70%, 90%, and 100%), for 15 min in each. The samples were then transferred to a mixture (1∶1, v/v) of ethanol and tertiary butanol, and pure tertiary butanol, for 20 min in each. After lyophilization and gold coating, the specimens were observed using a scanning electron microscope (Hitachi S-4800, Japan).

### Transmission Electron Microscope (TEM)

Preparation of the bacteria samples was conducted in the same manner as for SEM treatment. After fixing with 2.5% glutaraldehyde overnight, the bacterial pellets were washed three times with PBS and post-fixed with 1% osmium tetroxide in PBS for 2 h. The fixed bacterial cells were washed three times with PBS, and followed by dehydration for 15 min in a graded ethanol series (50%, 70%, 90%, and 100%). After placing in absolute acetone for 20 min, these samples were transferred to 1∶1 and 1∶3 mixture of absolute acetone and epoxy resin for 1 h in each, followed by transferring to pure epoxy resin for overnight. Ultrathin sections obtained by using ultramicrotome, were post-stained with uranyl acetate and lead citrate. Specimens were observed by transmission electron microscope (Hitachi H-7650, Japan).

### CD Analysis

Circular dichroism spectra of the peptides in different environments were obtained at 25°C by using a J-820 spectropolarimeter (Jasco, Tokyo, Japan). The peptide samples were recorded at a final concentration of 150 µM in 10 mM sodium phosphate buffer, pH 7.4 (mimicking the aqueous environment), 30 mM SDS micelles (mimicking the negatively charged prokaryotic membrane comparable environment, Sigma), and 50% TFE (mimicking the hydrophobic environment of the microbial membrane, Sigma). The samples were loaded in a rectangular quartz cell (0.1 cm path length), and the spectra were recorded at a scanning speed of 10 nm/min in the wavelength range of 190 to 250 nm.

## Results

### Antimicrobial Activities of the Peptides

MICs of the synthetic peptides against gram-negative and gram-positive bacteria are presented in Table 2. GI24 corresponding to the N-terminal of PMAP-36 displayed activity comparable to the full length peptide with MICs ranging from 1 to 4 µM, while no antimicrobial activity of PG12 (12-residue C-terminal fragment of PMAP-36) was observed for tested microorganisms. Further truncating from GI24, resulted in a significantly decrease or loss of antimicrobial activity, as demonstrated by GK12 and RI12, respectively.

**Table 2:** Antimicrobial activity of the peptides.

Peptide	MIC (µM)^a^						GM (µM)^b^
	*E. coli* ATCC25922	*E. coli*UB 1005	*S.* typhimuriumC77-31	*S. aureus*ATCC 29213	*S. aureus*ATCC 25923	*S. epidermidis*ATCC 12228	
PMAP-36	1	2	1	2	2	2	1.59
GI24	1	2	1	2	4	2	1.78
GK12	128	128	>128	>128	>128	>128	203.19
RI12	8	16	8	64	128	64	28.51
PG12	>128	>128	>128	>128	>128	>128	256
GI24-V3	4	4	4	4	4	2	3.56
GI24-V6	2	4	4	2	2	2	2.52
GI24-W23A	16	16	>128	128	128	128	71.84
GI24-W23K	32	16	>128	32	64	32	45.25
GI24-W23L	1	2	2	4	4	4	2.52
melittin	2	2	2	8	8	0.5	2.52

a Minimum inhibitory concentrations (MICs) were determined as the lowest concentration of the peptides that inhibited bacteria growth.

b The geometric mean (GM) of the MICs of the peptides against all four bacterial strains was calculated. When no antimicrobial activity was observed at 128 µM, a value of 256 µM was used to calculate the geometric mean.

The antimicrobial activity of the derivatives of GI24 were further determined and compared to GI24. As shown in Table 2, increasing the hydrophobicity of GI24 by substituting cationic residues (R or K) with hydrophobic residues (V) did not improved the antimicrobial activity of GI24-V3 or GI24-V6 with MICs against tested bacteria for 2 or 4 µM. The single site-substituting of W at position 23 of GI24 with A or K to give GI24-W23A or GI24-W23K resulted in a notable decrease in antimicrobial activities, especially in the case of GI24-W23A. The GM value of GI24-W23A was about 40 times higher than that of GI24. When the W was replaced by L, the antimicrobial activity of GI24-W23L recovered to the original level of inhibition observed for GI24.

### Hemolytic Activity of the Peptides

**Figure 2:**
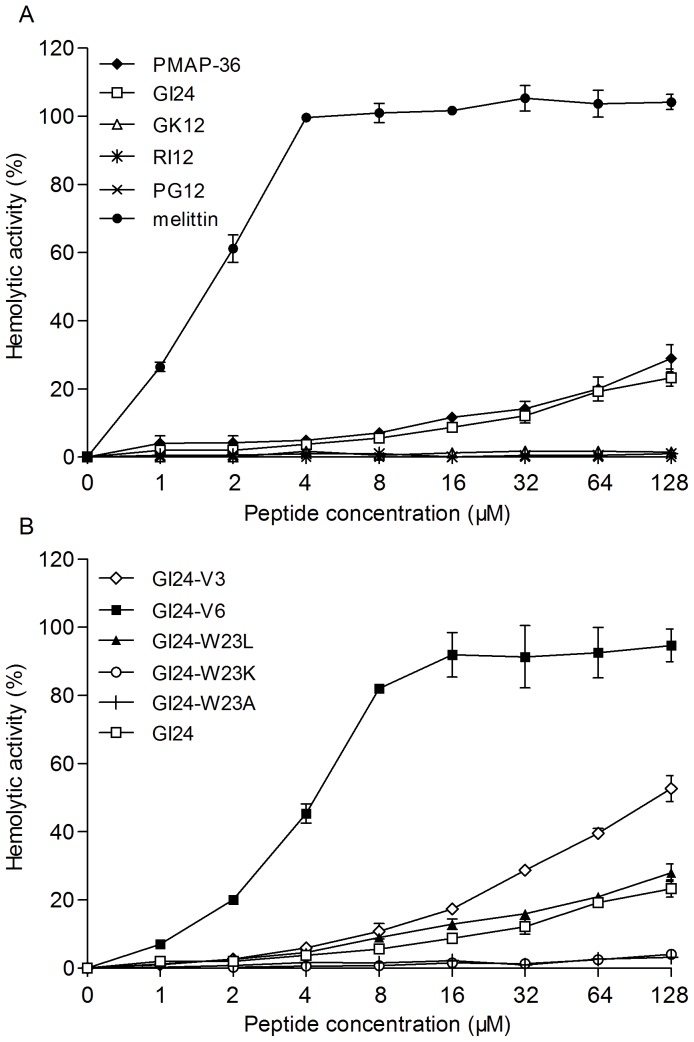
Hemolytic activities of the truncated peptides (A) and residue-substituted peptides (B) against fresh human erythrocytes. The release of hemoglobin was monitored with a Microplate autoreader by measuring the absorbance at 570 nm. Data are the averages of three independent experiments.

The hemolytic activity of the peptides against the highly sensitive human erythrocytes was determined as a measure of their toxicity to mammalian cells (Figure 2). GK12, RI12, PG12, GI24-W23K, and GI24-W23A displayed no hemolytic activity even at the maximum concentration of 128 µM. PMAP-36, GI24, and GI24-W23L showed slightly hemolytic activity in a dose-dependent manner. GI24-V3 and GI24-V6 demonstrated relatively high hemolytic activity at each concentration when compared to GI24. In contrast, melittin used as a control peptide caused complete hemolysis at a low concentration of 4 µM.

### Binding of Peptides to Model Membranes

The binding of peptides to lipid vesicles composed of PE/PG (7∶3, w/w) or PC/cholesterol (10∶1, w/w) was measured by monitoring the emission spectra of a tryptophan fluorophore (Table 3 and Figure S1). In Tris-HCl buffer, the maximum wavelengths of the peptides were approximately 350 nm, indicating the exposure of the tryptophan fluorophore to aqueous environment [28]. When the peptides were titrated to PE/PG phospholipid vesicles, a large blue shift was observed. However, little or no shift in wavelength was observed in PC/cholesterol phospholipid vesicles for PMAP-36 and GI24. These results suggested that the degree of insertion of the W into the zwitterionic vesicles was significantly less than that for vesicles with net negative charge. As a reference, melittin induced larger blue shifts in both PE/PG and PC/cholesterol phospholipid vesicles.

**Table 3:** Fluorescence spectroscopy parameters measured for the peptides in the presence and absence of PE/PG and PC/cholesterol vesicles.

Peptide	Fluorescence emission maxima (nm)			*K_SV_* b (M^−1^)		
	Buffer	PE/PG	PC/cholesterol	Buffer	PE/PG	PC/cholesterol
PMAP-36	348	336 (12^a^)	348 (0)	13.7	1.3	5.0
GI24	350	340 (10)	350 (0)	14.3	1.3	5.0
melittin	351	333(18)	335(16)	11.8	0.9	1.7

a Blue shift of emission maximum compared to Tris buffer.

b Stern-Vollmer constant *K_SV_* were calculated by the Stern-Vollmer equation: *F_0_/F* = 1+*K_SV_* (*Q*), where *Q* is the concentration of the quencher (acrylamide). Concentrations of the quencher were increased from 0.01 to 0.40 M. A smaller *K_SV_* value reflects a more protected W residue.

### Tryptophan Fluorescence Quenching by Acrylamide

To further investigate the extent of the burial of the W into phospholipids, a fluorescence quenching experiment was performed using a neutral quencher acrylamide [29]. A smaller *K_SV_* value reflects a more protected W residue. The results are presented in Table 3 and Figure S2. PMAP-36 and GI24 displayed same *K_SV_* values in the presence of PE/PG and PC/cholesterol phospholipid vesicles. In addition, the *K_SV_* values of PMAP-36 and GI24 in PE/PG vesicles were less than those in PC/cholesterol, demonstrating that the peptides were more protected in bacteria-mimicking cell membranes. The *K_SV_* value of melittin was smaller than PMAP-36 and GI24 in both PE/PG and PC/cholesterol phospholipid vesicles.

### Permeabilization of Outer Membranes

**Figure 3:**
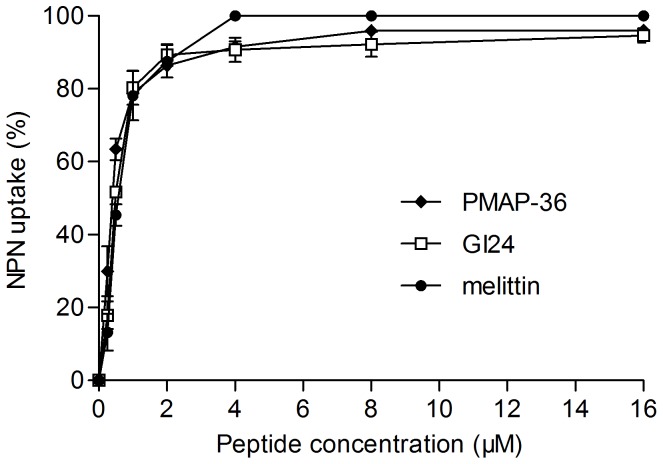
The uptake of NPN by *E. coli* UB1005 in the presence of different concentrations peptides. The ability of peptides to disrupt the outer membrane integrity of bacteria was evaluated with the fluorescent dye N-phenyl-1-naphthylamine (NPN). Changes in fluorescence were measured using an F-4500 fluorescence spectrophotometer at an excitation wavelength of 350 nm and an emission wavelength of 420 nm.

The outer membrane permeabilization of *E. coli* was determined by using the NPN uptake assay. NPN, a neutral hydrophobic fluorescent probe, is normally excluded by the outer membrane but exhibits increased fluorescence intensity when it partitions into the outer membrane. As shown in Figure 3, PMAP-36 and GI24 rapidly permeabilized the outer membrane of *E. coli* in a concentration-dependent manner as observed by an increase in NPN fluorescence. The peptides were able to permeabilize the outer membrane even at the concentrations lower than their MICs. The increase in fluorescence of PMAP-36 and GI24 was similar with that of melittin at the same concentrations.

### Depolarization of Inner Membranes

**Figure 4:**
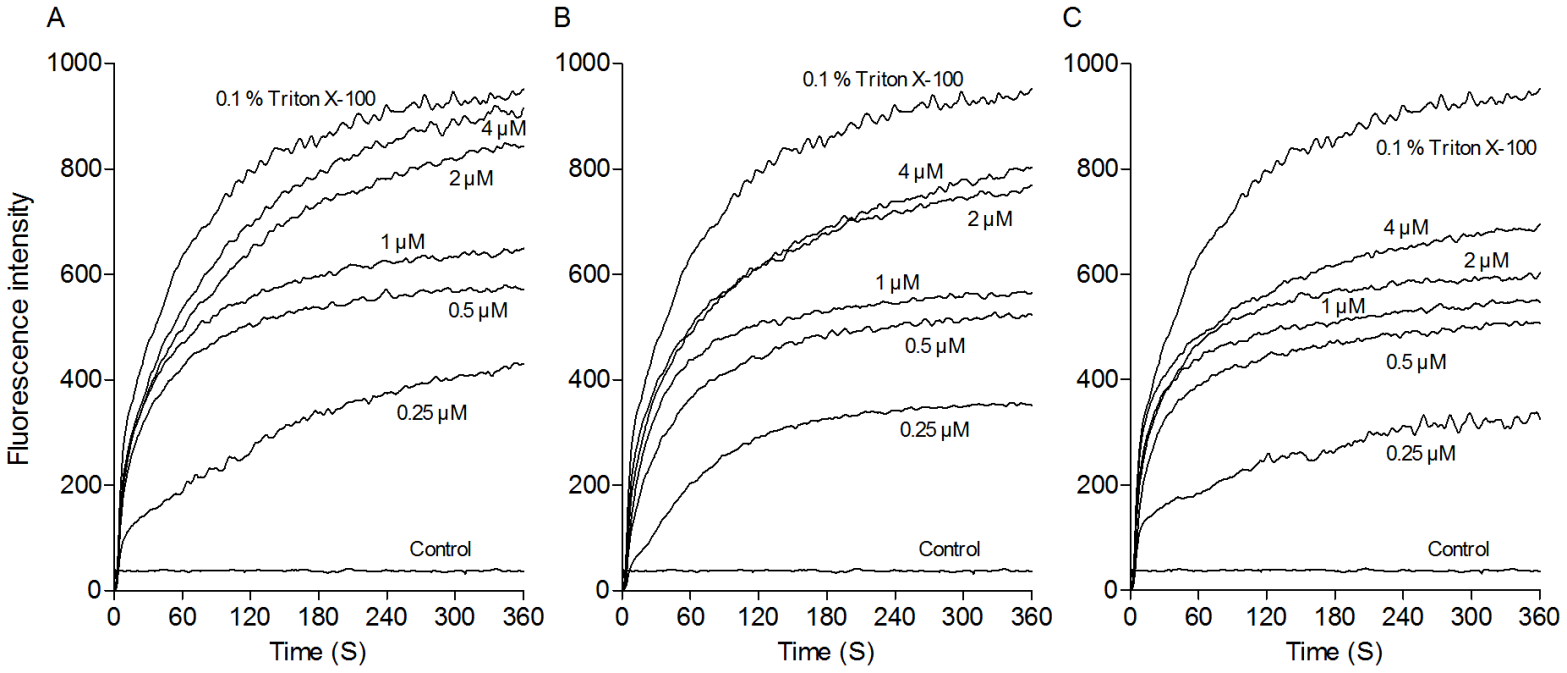
Cytoplasmic membrane depolarization of *E. coli* UB1005 by the peptides. The cytoplasmic membrane permeabilization of the peptides was measured using the membrane potential-sensitive dye, diSC_3_(5). Changes in fluorescence were recorded with an F-4500 fluorescence spectrophotometer at an excitation wavelength of 622 nm and an emission wavelength of 670 nm. (A) PMAP-36; (B) GI24; (C) melittin.

The membrane potential-sensitive dye diSC_3_(5) was used to evaluate the depolarization of the peptides on *E. coli* cytoplasmic membranes. Depolarization of the peptides was monitored over a period of 360 s (Figure 4). The results showed that three peptides PMAP-36, GI24, and melittin induced dose-dependent increases in diSC_3_(5) fluorescence, reflecting cytoplasmic membrane depolarization. PMAP-36 and GI24 were more effective and rapidly at permeabilizing the inner membrane than melittin at the same molar concentrations, especially when the peptide concentration is greater than their MIC. An immediate increase in fluorescence intensity was detected in 60 s after the addition of the peptides to *E. coli* suspensions.

### FACScan Analysis

**Figure 5:**
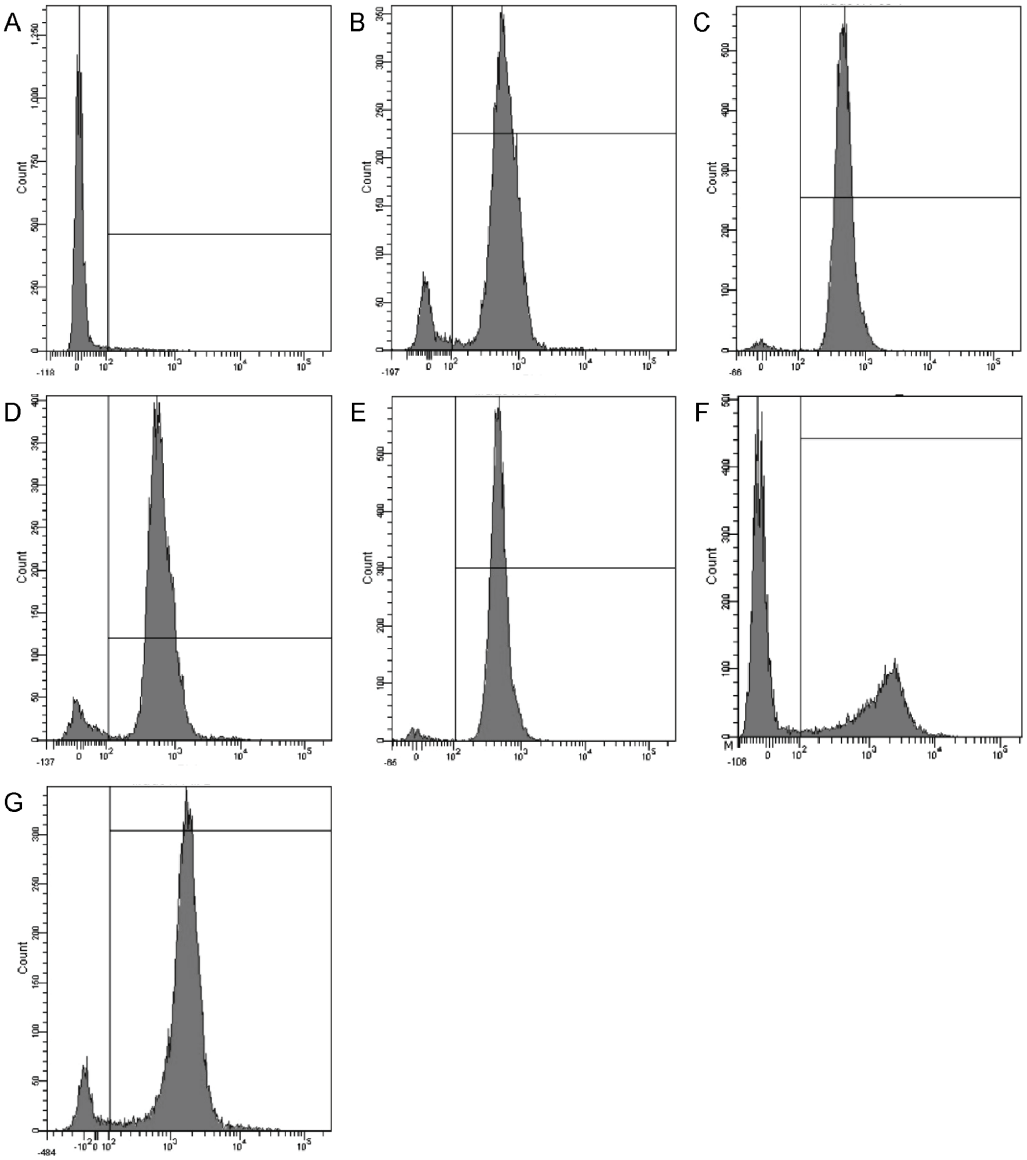
Flow cytometric analysis. The DNA intercalating dye propidium iodide (PI) was used to evaluate the cell membrane integrity via flow cytometry. The fluorescence intensity was monitored after treating with the peptides. Flow cytometry was performed using a FACScan instrument. (A) control, no peptide; (B) PMAP-36 (MIC, 1 µM); (C) PMAP-36 (2MIC, 2 µM); (D) GI24 (MIC, 1 µM); (E) GI24 (2MIC, 2 µM); (F) melittin (MIC, 2 µM); (G) melittin (2MIC, 4 µM).

The DNA intercalating dye propidium iodide (PI) was used to evaluate cell membrane integrity by flow cytometry. The fluorescence intensity indicates that the peptides induced the influx of PI into the cells. As shown in Figure 5, in control bacterial cells (no peptide), only 3.0% of the bacterial cells had PI fluorescence (Figure 5A). The majority of the bacterial cells (>90%) were labeled fluorescently after treatment with PMAP-36 and GI14 at MIC or 2MIC. However, melittin resulted in only 40.7% cells stained at MIC. A significant increase in staining ratio of melittin was observed when the peptide concentration increased from MIC to 2MIC.

### SEM

**Figure 6:**
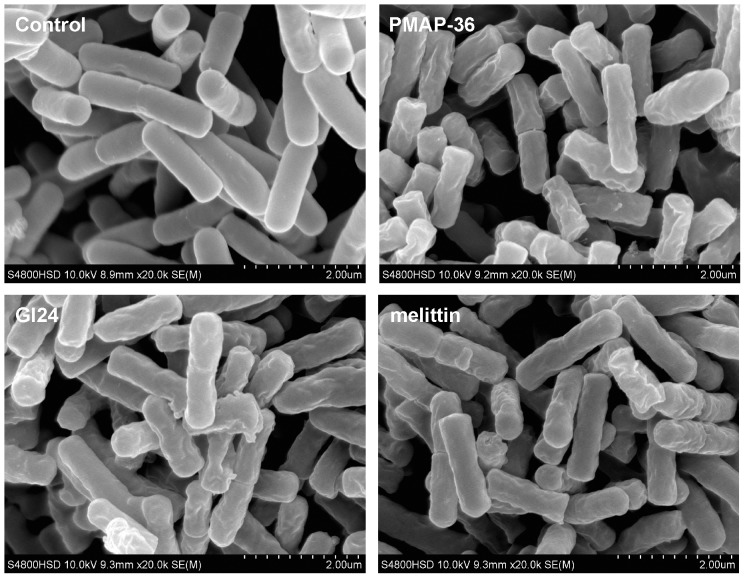
Scanning electron microscopic micrographs of *E. coli* treated with peptides. Bacteria were treated with peptides at MIC for 60 min. The control was done without peptides.

To directly observe cell morphological changes after peptides treatment, a SEM study was conducted. *E. coli* cells treated for 60 min with PMAP-36, GI24, and melittin at 1×MIC were visualized. As shown in Figure 6, treatment with all peptides induced membrane surface disruption in comparison to the control, which exhibited a bright and smooth surface. Bacterial cells treated with PMAP-36 and GI24 became surface roughening and corrugating, similar to the cells treated with melittin.

### TEM

**Figure 7:**
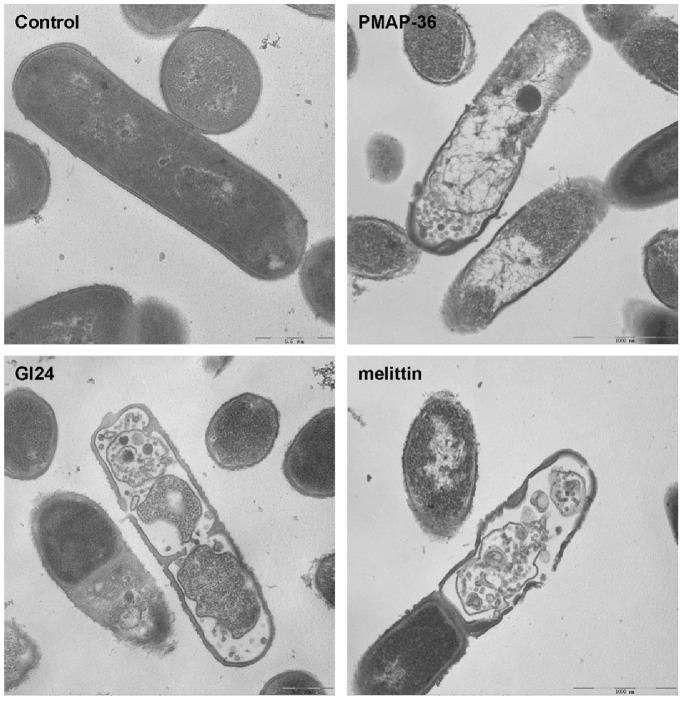
Transmission electron microscopic micrographs of *E. coli* treated with peptides. The bacteria cells were incubated with the peptides at MIC for 60 min at 37°C.

In addition to SEM, TEM was employed to study the membrane integrity and intracellular alteration of *E. coli* cells before and after treatment with the peptides. As shown in Figure 7, complete cell membrane and full intracellular contents were observed in the untreated *E. coli* cells. It can be seen that the treated bacteria cells by PMAP-36, GI24, and melittin, exhibited obvious cytoplasmic clear zones, and the integrity of the *E. coli* membrane was disrupted with visible pores.

### CD Analysis

**Figure 8:**
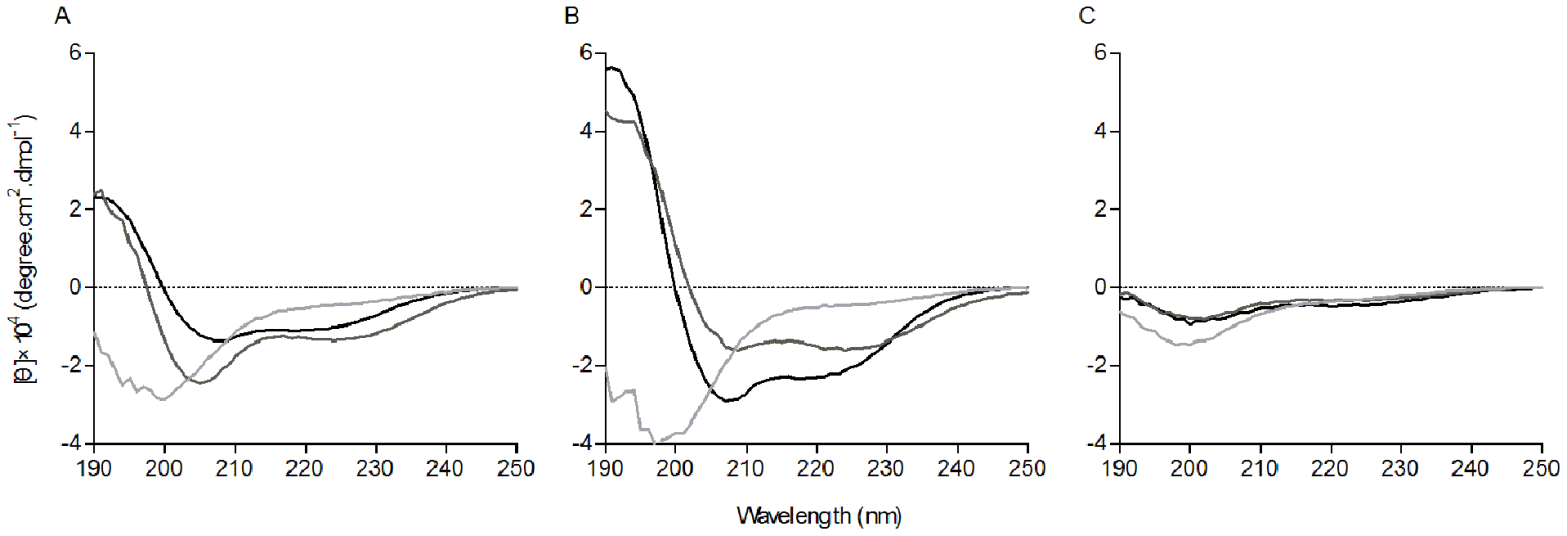
CD spectra of the peptides in aqueous solution (light grey), 30 mM SDS micelles (dark grey), and 50% TFE (black). The circular dichroism spectra were measured at 25°C using a J-820 spectropolarimeter. The samples were loaded in a rectangular quartz cell (0.1 cm path length), and the spectra were recorded at a scanning speed of 10 nm/min in the wavelength range of 190 to 250 nm. (A) PMAP-36; (B) GI24; (C) PG12.

Circular dichroism spectroscopy was performed for the peptides in sodium phosphate buffer, 30 mM SDS, and 50% TFE (Figure 8). All of the peptides formed random coil structures in aqueous solution as indicated by the presence of a strong minimum peak near 200 nm. When 30 mM SDS and 50% TFE were mixed with PMAP-36 and GI24, an increase in the mean residue ellipticity at 208 and 222 nm was observed, consistent with the formation of an α-helix [30]. The conformation of PG12 in the presence of SDS or TFE was the same as that in buffer, with a random coil structure.

## Discussion

The truncation and residue-substitution of natural antimicrobial peptide is considered an effective method for developing a candidate of antimicrobial peptides with fewer residues. Several studies have demonstrated that it is possible to truncate the naturally occurring peptide to less than half its length and retain antimicrobial and other functions [31], [32]. Studies on cathelicidin BMAP-27 and its truncated derivative indicated that the α-helical fragment BMAP-18 was almost equally active to the full length peptide [33], [34]. Similar results were found for the peptide in this study. The deletion of C-terminal hydrophobic tail did not affect the antimicrobial activity of GI24, the N-terminus truncated derivative of PMAP-36. GI24 displayed high antimicrobial activity that was comparable to that of parental PMAP-36. However, the antimicrobial activity of GK12 and RI12 further truncated peptides from PMAP-36 was significantly reduced as compared to parental peptide. PG12, the C-terminal sequence of PMAP-36 with 12 residues, displayed no antimicrobial activity against all tested bacteria, which may be due to the absence of cationic amino acids. These results indicated that truncated derivative (GI24) may retain complete antimicrobial activity of parental peptide (PMAP-36).

Furthermore, according to the helical wheel projection, we noticed that GI24 displayed an unusually wide cationic polar face (Figure 1). Previous studies have shown that increasing hydrophobicity in a certain range contributed to improve antimicrobial activity [35]. For this purpose, two analogs of GI24 were synthesized by substituting R or K with V. The net positive charge decreased from +14 (GI24) to +11 (GI24-V3) and +8 (GI24-V6), and the hydrophobicity increased from −2.81 (GI24) to −1.05 (GI24-V3) and 0.69 (GI24-V6). Antimicrobial assay showed that the hydrophobic increase did not improve the antimicrobial activity as we expected. On the contrary, the antimicrobial activity of GI24-V3 and GI24-V6 was slightly reduced as compared to GI24. This may be due to the low solubility of high hydrophobic peptides. In addition, compared with PMAP-36 (1–20), a truncated N-terminal derivative with 20 residues reported by Storici et al [18], we noticed that GI24 displayed approximately 3–24 fold higher antimicrobial activity across the bacterial species. Actually, we also synthesized the N-terminal sequence of PMAP-36 with 20 residues. Antimicrobial assay showed that the peptide GRFRRLRKKTRKRLKKIGKV displayed extremely low antimicrobial activity against six bacterial strains tested (data not shown). The difference of antimicrobial activity indicated that residues 21–24 (LKWI) are important for the antimicrobial activity of GI24. Among the four residues (LKWI), the W attracted our attention. It was reported that W with bulky side chain ensured more efficient interaction of peptides with the bacterial membrane [36], [37]. We conjectured that the W at position 23 of GI24 may play a crucial role in antimicrobial activity, and the lost of W may be an important reason for the reduced antimicrobial activity of PMAP-36 (1–20). So, to investigate the contribution of W at position 23 of GI24 on the antimicrobial activity, a series of W-substituted mutants were developed by substituting W with A, K, and L. Antimicrobial assay showed that the antimicrobial activity of GI24-W23A and GI24-W23K against gram-negative and gram-positive bacterial was significantly reduced. When the W of GI24 was replaced with L, the antimicrobial activity of GI24-W23L was recovered to a level similar to GI24. We noticed that the antimicrobial activity of these peptides seem to be well correlated with their mean hydrophobicity (Table 1). GI24 and GI24-W23L shared the same mean hydrophobicity (−2.81), which was higher than that of GI24-W23A (−3.26) and GI24-W23K (−3.63). These results suggested that the hydrophobicity of an amino acid at position 23 played an important role in determining the level of antimicrobial activity of GI24 rather than the bulky side chain of W. In addition, both PMAP-36 and GI24 displayed strong resistance to salts (Table S1).

The hemolytic activity of the peptides against highly sensitive human erythrocytes is an important indicator of the toxicity to mammalian cells. All 12-residue peptides displayed no hemolysis at the tested concentrations. The hemolytic activity of the 24-residue derivatives was related to the hydrophobicity, especially for high hydrophobic GI24-V3 and GI24-V6 which caused 50% hemolysis at 128 µM and 8 µM, respectively. Chou et al reported that high hydrophobicity and amphipathicity (hydrophobic moment) are more correlated with increased hemolytic activity rather than antimicrobial activity [38]. Our results indicated that both size and hydrophobicity modulated hemolytic activity, and short peptides represented more performance committed to decrease the hemolytic activity. GI24 did not cause 50% hemolysis even at the highest determination concentration (128 µM), which was at least 60 times larger than its MICs.

The CD spectra exhibited that all peptides displayed typical random coil structure in aqueous solution. The addition of TFE and SDS induced a conformational change in PMAP-36 and GI24 consistent with the formation of α-helical structure, which was consistent with our hypothesis and confirmed the previous reports [18], [19]. After the initial electrostatic adsorption, AMPs aggregate on the surface of bacterial cell and correct orientation according to the plane of binding, following with the partitioning of the peptide to the membrane and the α-helical amphipathic structure transition. This conformational transformation is the key feature for AMPs to partition in bacterial cell membranes, which ultimately leads to bacterial cell death [39].

To elucidate the interaction of the peptides with phospholipid membranes, the fluorescence emission and quenching of W residue were monitored. Blue shift of emission maximum and Stern-Vollmer constant *K_SV_* value reflect the different cell selectivity of the peptides. PE/PG (7∶3, w/w) or PC/cholesterol (10∶1, w/w) SUVs were prepared to simulate negatively charged or zwitterionic membranes. The shift of the maximum emission in the presence of the lipid vesicles is because of the sensitivity of the W to the polarity of its environment [40]. Both PMAP-36 and GI24 displayed larger blue shifts and smaller *K_SV_* values in the negatively charged PE/PG vesicles than in the zwitterionic PC/cholesterol vesicles, suggesting that the tryptophan of the peptides insert or penetrate bacteria-mimicking membranes more deeply than eukaryote-mimicking membranes [41]. The selective membrane interaction of PMAP-36 and GI-24 corresponded well with the high antimicrobial activity and weak hemolytic activity.

Results of the peptide-lipid interactions suggested that membrane permeation may play a key role in the antimicrobial activity of PMAP-36 and GI24. Thus, the outer and cytoplasmic membrane permeability assays were performed to detect the target site of the peptides. An increase in the NPN fluorescence of the peptides indicated that all of the peptides mediated NPN uptake across the outer membrane in a dose-dependent manner. Then, the ability of the peptides to depolarize cytoplasmic membrane was assayed by using the membrane potential-sensitive dye diSC_3_(5), which concentrates in the cytoplasmic membrane under the influence of the membrane potential resulting in a self-quenching of fluorescence. If the cytoplasmic membrane is disrupted or forms channels, the fluorescent probe will be released into the medium, causing an increase in fluorescence [42]. PMAP-36 and GI24 induced different extent of the depolarization to cytoplasmic membrane in a dose-dependent manner. Consistent with previous study [19], the membrane permeability experiments suggested that PMAP-36 and GI24 targeted the cell membrane, similar to melittin, a membrane-active peptide. PMAP-36, GI24, and melittin caused similar NPN uptake (Figure 3), and the increased fluorescence intensity of GI24 and melittin in the inner membrane permeabilization (Figure 4) was slight less than that of PMAP-36. However, PMAP-36, GI24, and melittin displayed same MIC against *E. coli* UB1005, indicating that the difference in the antimicrobial activity of the peptides is likely due to the difficulties in permeabilizing the outer membrane, and not the inner membrane. The outer membrane of gram-negative bacteria forms an effective permeability barrier composed of a stable complex of lipopolysaccharide (LPS) and divalent cations. The initial process of peptide-membrane interaction was considered the displacement of divalent cations that stabilize adjacent polyanionic LPS molecules [43]. With 14 net positive charges, PMAP-36 and GI24 can associate readily with anionic membranes via electrostatic interactions. Following initial attachment, the peptides aggregate and reach a threshold concentration, which enables productive action. At this concentration, peptides begin to rearrange and alter pathogen permeability via generally accepted mechanisms [1]. Then, antimicrobial peptides insert into the hydrophobic core of the membrane and promote the formation of transmembrane pore-like structures [44], [45].

To further assess whether the peptides damaged the bacterial cell membrane, we determined PI staining of nucleic acids as an indicator of cell death. In addition, scanning electron microscopy and transmission electron microscope assays were employed to directly observe the cell morphological changes as well as the integrity of the *E. coli* membrane. FACScan analysis indicated that treatment of *E. coli* cells with the peptides enhanced uptake of PI, suggesting that the bacterial cell membrane were disrupted. The surface roughening and significant rupture of cell membranes and the release of cellular contents of *E. coli* treated with both PMAP-36 and GI24 were observed by SEM and TEM. These data further confirmed that both PMAP-36 and GI24 kill bacteria via membrane disruption, and roughening and corrugating observed on the cell membrane surface is probably due to the leakage of intracellular contents.

Previous studies have shown that melittin kills bacterial via a toroidal-pore mechanism [46]–[49]. Furthermore, increased accumulation of certain peptides such as melittin may lead to a detergent-like disintegration of membrane via a carpet mechanism [50], [51]. Sato and Feix also pointed out that different membrane-active mechanism need not be mutually exclusive, one process may represent an initial or intermediate step and another may be its consequence [52]. In this study, the membrane-active experiments revealed that PMAP-36 and GI24 damaged the cell membrane in a manner similar to melittin. The peptides rapidly permeabilized the out and inner membrane of *E. coli* even at the concentrations lower than their MICs (Figure 3 and 4). This membrane-permeabilizing behavior was estimated by the torodal pore-formation. However, visible pores with large diameter were observed in the *E. coli* membrane treated with the peptides by TEM (Figure 7). The diameter of the pore in the membrane treated with the peptides was larger than the pore sizes of melittin (1.5 to 5 nm) reported in toroidal-pore mechanism [46]–[48]. So, we conjectured that PMAP-36 and GI24 damaged the cell membrane by a detergent-like carpet mechanism at a high peptide concentration similar to melittin.

In summary, the results presented here demonstrate that the antimicrobial activity of the porcine myeloid antimicrobial peptide PMAP-36 was not affected by the truncation of the 12-residue C-terminal tail. GI24 displayed strong antimicrobial activity and weak hemolytic activity. The hemolysis assay showed that the hemolytic activity of long peptides was more sensitive to the increase of hydrophobic as compared to short peptides. In addition, the crucial site of GI24 was identified through single site-mutation. Then, the membrane-active mechanisms were determined by using lipid vesicles and whole bacteria. The preference of PMAP-36 and GI24 for binding to negatively charged phospholipids over zwitterionic phospholipids, which led to greater cell selectivity. The screened peptide GI24 was shown to permeabilize the outer membrane of bacterial cells and depolarize the cytoplasmic membrane, thus damaging the membrane integrity and causing intracellular content leakage, which leads to cell death. The findings reported in this study supported that truncation is an effective strategy for developing novel antimicrobial peptides, and GI24 could be a promising antimicrobial agent for clinical application.

## Supporting Information

Figure S1 **Tryptophan fluorescence emission spectra of PMAP-36 (A), GI24 (B), and melittin (C) in the buffer or in the presence of PE/PG or PC/cholesterol liposomes.**
(TIF)

Figure S2 **Stern–Volmer plots for the quenching of Trp fluorescence of PMAP-36 (A), GI24 (B), and melittin (C) by acrylamide in the buffer or in the presence of PE/PG or PC/cholesterol liposomes.**
(TIF)

Table S1 (DOC)
