# Supplementary figures and images for: Antimicrobial Properties and Membrane-Active Mechanism of a Potential α-Helical Antimicrobial Derived from Cathelicidin PMAP-36
Source: PLoS One. 2014 Jan 21;9(1):e86364. doi: 10.1371/journal.pone.0086364 (PMC3897731; doi:10.1371/journal.pone.0086364)

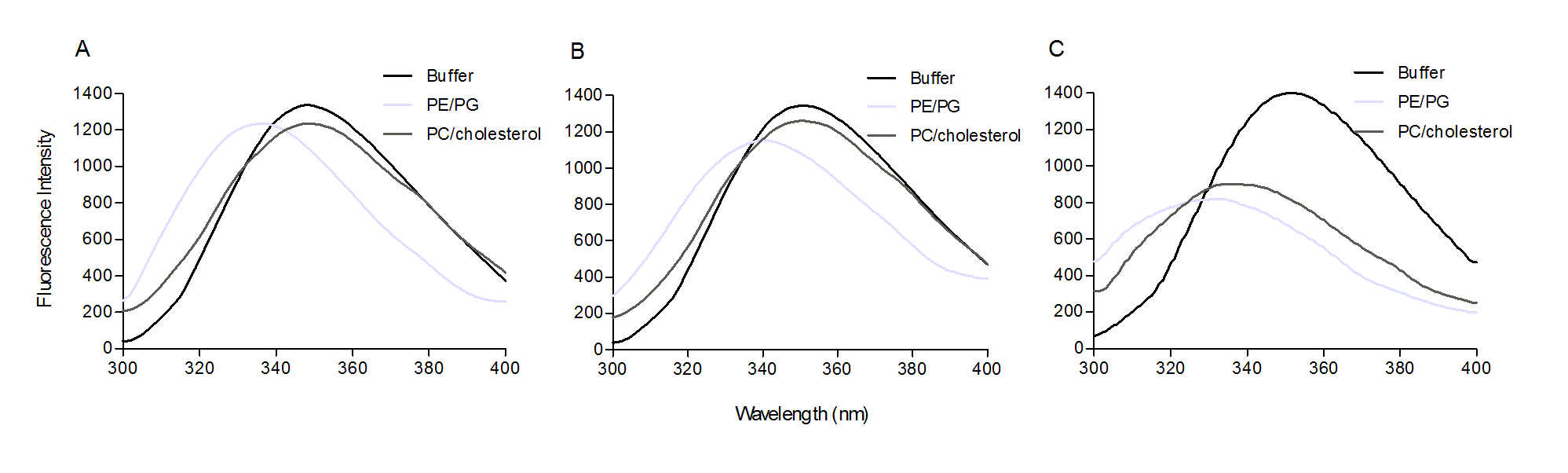

Supplement: Figure S1 — Tryptophan fluorescence emission spectra of PMAP-36 (A), GI24 (B), and melittin (C) in the buffer or in the presence of PE/PG or PC/cholesterol liposomes. (TIF) [file pone.0086364.s001.tif]

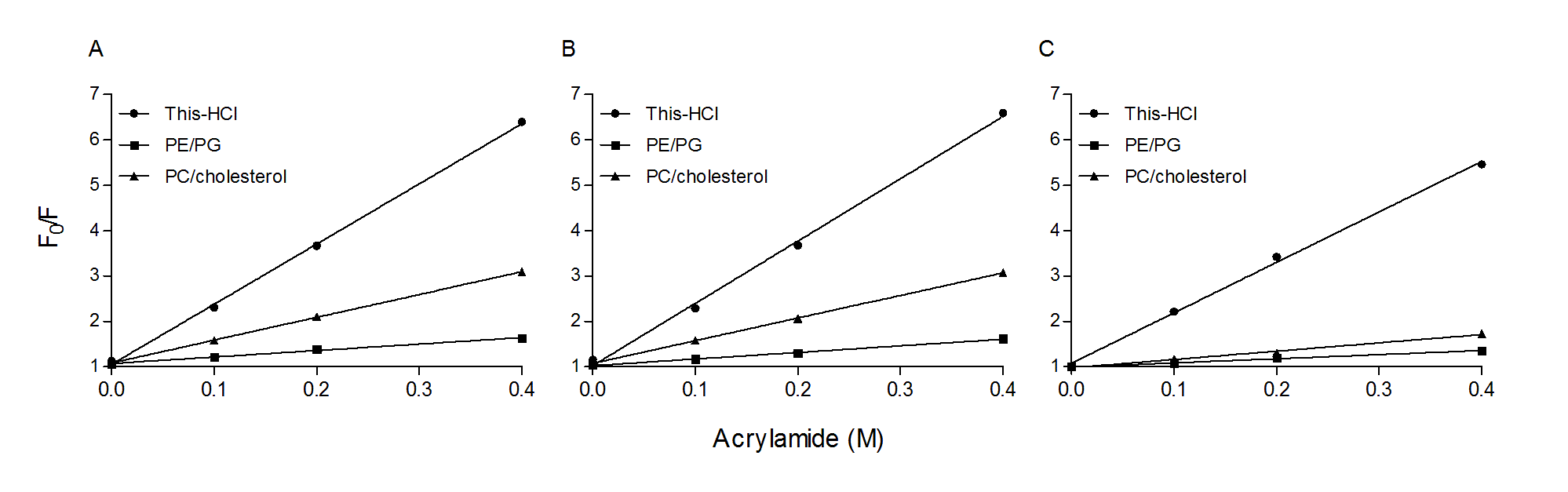

Supplement: Figure S2 — Stern–Volmer plots for the quenching of Trp fluorescence of PMAP-36 (A), GI24 (B), and melittin (C) by acrylamide in the buffer or in the presence of PE/PG or PC/cholesterol liposomes. (TIF) [file pone.0086364.s002.tif]
