# Supplementary material for: Antimicrobial Properties and Membrane-Active Mechanism of a Potential α-Helical Antimicrobial Derived from Cathelicidin PMAP-36
Source: PLoS One. 2014 Jan 21;9(1):e86364. doi: 10.1371/journal.pone.0086364 (PMC3897731; doi:10.1371/journal.pone.0086364)
Supplement: Table S1 — (DOC) [file pone.0086364.s003.doc]

**Table S1. Effects of salts on antimicrobial activity of the peptides**

| **Treatment** | **MIC (μM)** | |
| --- | --- | --- |
|  | **PMAP-36** | **GI24** |
| Without treatment | 1 | 1 |
| NaCl (50 mM) | 1 | 1 |
| NaCl (100 mM) | 1 | 2 |
| NaCl (150 mM) | 2 | 2 |
| MgCl_2_ (1 mM) | 2 | 2 |
| CaCl_2_ (1 mM) | 1 | 1 |
